# Momentary Depressive Feeling Detection Using X (Formerly Twitter) Data: Contextual Language Approach

**DOI:** 10.2196/49531

**Published:** 2023-11-27

**Authors:** Ali Akbar Jamali, Corinne Berger, Raymond J Spiteri

**Affiliations:** 1 Department of Computer Science University of Saskatchewan Saskatoon, SK Canada

**Keywords:** depression, momentary depressive feelings, X (Twitter), natural language processing, lexicon, machine learning, transfer learning

## Abstract

**Background:**

Depression and momentary depressive feelings are major public health concerns imposing a substantial burden on both individuals and society. Early detection of momentary depressive feelings is highly beneficial in reducing this burden and improving the quality of life for affected individuals. To this end, the abundance of data exemplified by X (formerly Twitter) presents an invaluable resource for discerning insights into individuals’ mental states and enabling timely detection of these transitory depressive feelings.

**Objective:**

The objective of this study was to automate the detection of momentary depressive feelings in posts using contextual language approaches.

**Methods:**

First, we identified terms expressing momentary depressive feelings and depression, scaled their relevance to depression, and constructed a lexicon. Then, we scraped posts using this lexicon and labeled them manually. Finally, we assessed the performance of the Bidirectional Encoder Representations From Transformers (BERT), A Lite BERT (ALBERT), Robustly Optimized BERT Approach (RoBERTa), Distilled BERT (DistilBERT), convolutional neural network (CNN), bidirectional long short-term memory (BiLSTM), and machine learning (ML) algorithms in detecting momentary depressive feelings in posts.

**Results:**

This study demonstrates a notable distinction in performance between binary classification, aimed at identifying posts conveying depressive sentiments and multilabel classification, designed to categorize such posts across multiple emotional nuances. Specifically, binary classification emerges as the more adept approach in this context, outperforming multilabel classification. This outcome stems from several critical factors that underscore the nuanced nature of depressive expressions within social media. Our results show that when using binary classification, BERT and DistilBERT (pretrained transfer learning algorithms) may outperform traditional ML algorithms. Particularly, DistilBERT achieved the best performance in terms of area under the curve (96.71%), accuracy (97.4%), sensitivity (97.57%), specificity (97.22%), precision (97.30%), and *F*_1_-score (97.44%). DistilBERT obtained an area under the curve nearly 12% points higher than that of the best-performing traditional ML algorithm, convolutional neural network. This study showed that transfer learning algorithms are highly effective in extracting knowledge from posts, detecting momentary depressive feelings, and highlighting their superiority in contextual analysis.

**Conclusions:**

Our findings suggest that contextual language approaches—particularly those rooted in transfer learning—are reliable approaches to automate the early detection of momentary depressive feelings and can be used to develop social media monitoring tools for identifying individuals who may be at risk of depression. The implications are far-reaching because these approaches stand poised to inform the creation of social media monitoring tools and are pivotal for identifying individuals susceptible to depression. By intervening proactively, these tools possess the potential to slow the progression of depressive feelings, effectively mitigating the societal load of depression and fostering improved mental health. In addition to highlighting the capabilities of automated sentiment analysis, this study illuminates its pivotal role in advancing global public health.

## Introduction

Mental health is an essential aspect of people’s overall well-being and daily functioning. According to the World Health Organization [1], approximately 25% of the global population experiences a mental health condition at some point in their life, making mental disorders a significant public health concern. Mental health conditions can have substantial socioeconomic impacts on individuals and society, including reduced quality of life and workforce productivity and increased health care costs. Accordingly, it is vitally important to prioritize and address mental health conditions by adopting effective strategies to curb their prevalence [2].

Subthreshold depression, or momentary depressive feelings, refers to depression symptoms that are not severe enough to be considered as a major depressive disorder [3]. Despite not meeting the criteria for a depression diagnosis, momentary depressive feelings can still significantly impact an individual’s daily life and well-being. Frequent or prolonged experiences of momentary depressive feelings can be a sign of the development of depression [4-6] and can lead to decreased energy, loss of interest in activities, and persistent low mood [7-9]. Mitchell et al [10] found that people with subthreshold depression reported higher levels of disability and decreased quality of life compared to those not reporting any symptoms.

Early detection of momentary depressive feelings is important for an individual’s mental health and well-being because it allows identifying individuals who may be at a higher risk of developing depression [11,12] and lets them address these feelings before they escalate into a more severe form of depression [6,13]. Detecting momentary depressive feelings can also provide important information for researchers and mental health professionals, leading to a better understanding of the nature of depression, and can be used to provide preventative interventions and support to help individuals maintain their mental health. However, it is worth noting that depression is a complex condition with multiple causes, and the detection of momentary depressive feelings should be considered in conjunction with a comprehensive evaluation of an individual’s overall mental health [14].

Momentary depressive feelings can be detected through different standard methods including self-report measures, behavioral observations, and physiological measures [15]. Self-report measures ask individuals to reflect on their current mood and symptoms, whereas behavioral observations involve observing and recording an individual’s behavior and facial expressions. Physiological measures, such as measuring cortisol levels, heart rate variability, and skin conductance, can also provide insight into an individual’s emotional state [16].

Momentary depressive feelings are often accompanied by distinctive linguistic patterns, allowing us to understand an individual’s emotional state and cognitive processes. One prevalent symptom is the expression of negative sentiment and emotion [17]. People with momentary depressive feelings or who are in depression frequently use language dominated by a pessimistic lexicon, conveying feelings of hopelessness, sadness, and despair. This linguistic tendency reflects their internal emotional turmoil and offers an insight into the depth of their distress. Another linguistic hallmark is the increase in self-referential language. The excessive use of first-person pronouns such as “I” or “me” suggests a potential focus on one’s own experiences and an emphasis on the self [18,19]. The study of these linguistic symptoms within contextual contents offers promising avenues for the detection of momentary depressive feelings using advanced methods. Contextual approaches have demonstrated outstanding ability in discerning linguistic markers of depression. These methods consider not only individual words but also the surrounding context, enabling a more accurate interpretation of the intended meaning.

One prominent opportunity in this domain involves sentiment analysis. Sentiment analysis attempts to gauge the emotional tone of the text. Contextual approaches for sentiment analysis such as Valence Aware Dictionary for Sentiment Reasoning (VADER) rely on predefined sentiment scores assigned to words. These models can swiftly identify texts with predominantly negative sentiments [20]. However, their generic lexicons might not capture the nuanced depressive expressions. Machine learning (ML) techniques such as support vector machines and decision trees have also been applied to classify depressive textual content. These models learn to differentiate between depressive and nondepressive contents by extracting features from the text, including n-grams and linguistic patterns [21]. Yet, these methods can struggle with complex contextual cues inherent in sentiment discourse. Subsequently, more sophisticated models have emerged using contextual methods such as Word2Vec and FastText to discover semantic relationships within text. These models offer the advantage of representing words in context, which is vital for detecting subtler depressive symptoms [22]. Similarly, deep learning architectures such as convolutional neural network (CNN) and long short-term memory network have been used to capture sequential dependencies in text, enhancing the understanding of the temporal progression of depressive feelings [23,24].

In recent years, the advent of pretrained language models such as Bidirectional Encoder Representations From Transformers (BERT) [25] has revolutionized the application of natural language processing (NLP) [26], including depressive feeling detection. NLP offers promising alternative methods for the detection of momentary depressive feelings using social media, such as Facebook, Instagram, and X (formerly Twitter), where individuals can broadcast their thoughts and feelings in real time [27]. NLP can be used to identify patterns in language and sentiment to recognize specific language and behavioral markers that may be indicative of depression. Sentiment analysis can be used to determine the underlying emotional tone and identify individuals at risk of depression using large-scale data sets. Numerous studies have applied sentiment analysis and text classification in the area of mental health [28-30] to detect effectively depressive feelings and depression [31-36].

X has emerged as a popular social media platform for mental health research due to its vast and diverse text-based data. Its real-time nature makes it particularly well-suited for studying a variety of mental disorders such as dementia [37], depression [38], Alzheimer disease [39], and schizophrenia [40]. The analysis of X data allows the detection of momentary mood changes and depressive feelings, offering a unique opportunity for identifying individuals who may be at risk of developing depression. X data also provide a chance to investigate the expression of mental disorders and develop novel ML-based methods for detecting other mental disorders.

The primary objective of this study is to develop contextual language approaches and assess their effectiveness in detecting momentary depressive feelings in posts. With the aid of large-scale data and advanced NLP techniques, this study aims to analyze linguistic features and patterns in posts to detect momentary depressive feelings. This study has the potential to provide valuable insights into the relationship between language and mental health. Additionally, it may contribute to the development of tools for the early detection and prevention of depression.

## Methods

### Study Design

**Figure 1:**
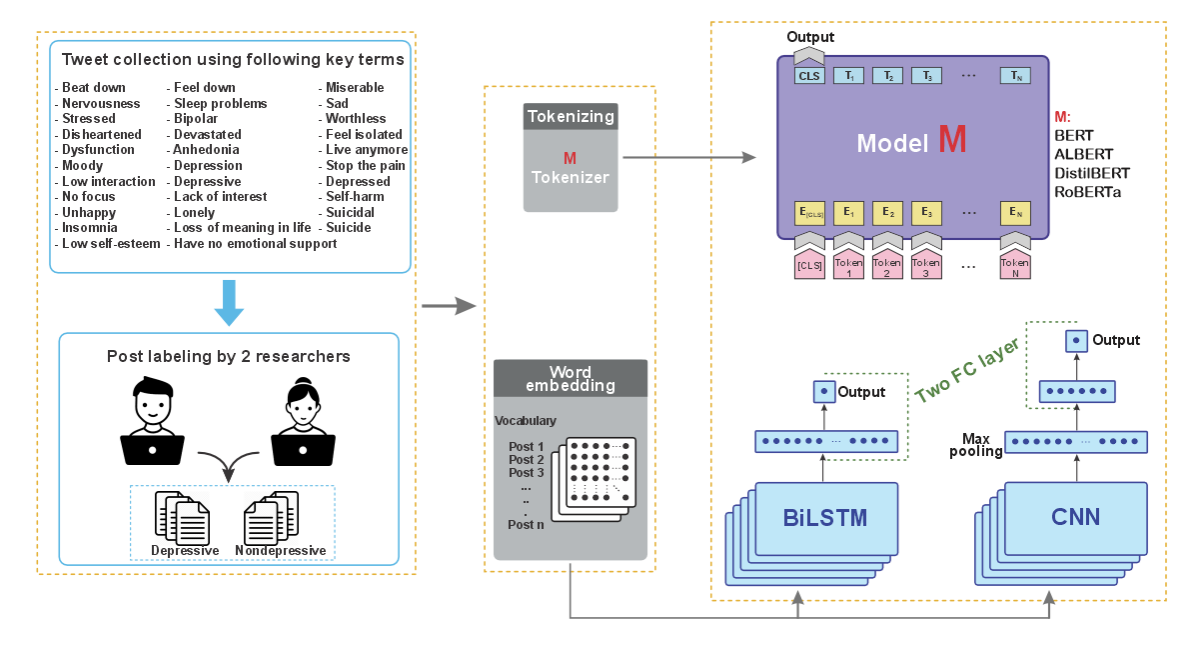
The workflow of data collection and architecture of the proposed detection approaches. ALBERT: A Lite BERT; BERT: Bidirectional Encoder Representations From Transformers; BiLSTM: Bidirectional Long Short-term Memory; CLS: classification tasks; CNN: Convolutional Neural Network; DistilBERT: Distilled BERT; FC: fully connected; RoBERTa: Robustly Optimized BERT Approach.

The overview and workflow of this study are presented in Figure 1. First, we identified and collected words expressing depression. We then scraped and manually labeled posts. Next, the posts in our data set were preprocessed and cleaned. Finally, state-of-the-art NLP algorithms were used for the detection of momentary depressive feelings in posts. This study was performed using Python (version 3.9; Python Software Foundation) and R (R Foundation for Statistical Computing) programming languages with different packages. The data collection, preparation, and ML algorithms used in this study are discussed in the following sections.

### Data Collection and Preparation

#### Overview

Contextual language approaches have been found to be effective in processing large text data sets, making them appropriate for text-based tasks such as sentiment analysis and for determining whether individual posts may express momentary depressive feelings. Accordingly, in order to collect appropriate data, we first need to construct a suitable lexicon and then use it to scrape for appropriate posts. The data are then manually labeled in preparation for training the ML algorithms.

#### Lexicon Construction

In the process of lexicon construction, we carefully examined and reviewed major studies that delve into the correlation between social media and depression, aiming to compile essential terms for our depression lexicon [41,42]. We specifically focused on key terms relevant to depressive feelings and collected a variety of these terms as previously highlighted in relevant research. Following the elimination of any redundant terms, we arrived at a total of 41 distinct key terms within our lexicon. Recognizing that the quality of the lexicon is pivotal for accurate contextual language analysis, 2 researchers (AAJ and CB) assessed the relevance of each term in our lexicon to depressive feelings using a 5-point scale, where a rating of 5 indicates significant relevance. In this study, we elected to only incorporate key terms with a score of 3 or higher. By applying this criterion, the lexicon was refined to contain 32 terms, as illustrated in Textbox 1.

**The lexicon for momentary depressive feeling detection.**

Included depressive feeling lexicons (n=32)

- Beat down, nervousness, stressed, disheartened, dysfunction, moody, low interaction, no focus, unhappy, insomnia, low self-esteem, feel down, have no emotional support, sleep problems, bipolar, devastated, anhedonia, depression, depressive, lack of interest, lonely, miserable, sad, worthless, feel isolated, live anymore, stop the pain, depressed, loss of meaning in life, self-harm, suicidal, and suicide

Excluded depressive feeling lexicons (n=9)

- Instability, imbalance, broken, disillusioned, emotional, uneasiness, disturbed, anxiety, and fatigue

#### Post Scraping

The *Twint* package was used to collect posts using a constructed lexicon and key terms. *Twint* is a Python package that allows scraping posts, followings, followers, likes, etc, from X without using X’s application programming interface. It provides several customization options for searching specific posts based on keywords, location, language, date, and more. Unlike the X application programming interface, *Twint* does not have any rate limits or access restrictions, making it possible to scrape large amounts of data. posts posted between January 1, 2022, and December 30, 2022, were extracted.

#### Post Labeling

Two researchers (AAJ and CB) labeled posts as expressing or not expressing momentary depressive feelings. To determine intercoder reliability, the researchers read and scored the same 100 posts on a 5-point scale, with 5 indicating significant relevance to momentary depressive feelings, and achieved intercoder reliability of 86% based on the percent agreement method [43]. Each researcher was then given 2000 posts to label. For binary classification, out of 4000 labeled posts, 1840 posts that received a score of 3 or higher were used as positive samples. To construct a balanced data set, 1840 negative samples (posts not expressing momentary depressive feelings) were randomly selected, and a final data set was constructed consisting of 3680 samples with an equal number of positive and negative samples. For multilabel classification, all labeled posts in 5 categories on a scale of 1 to 5 were included.

#### Preprocessing

Given that posts frequently contain misspelled words, irrelevant characters, emoticons, and unconventional syntax—considered noise in NLP—we implemented various preprocessing steps on our data set before training the algorithms. Key preprocessing steps included:

1. Filtration: Removing punctuations, emoticons, duplicates, replies, URLs, and HTML links
2. Tokenization: Breaking a phrase or sentence into individual words called tokens
3. Lowercasing: Converting all uppercase letters to lowercase and ensuring consistent word vectors across multiple instances of the same word
4. Lemmatization: Removing inflectional parts in a word or converting the word into its base form
5. Stemming: Removing prefixes or suffixes from words to obtain their root form
6. Removing stop words: Articles, prepositions, and pronouns (eg, the, in, a, an, and with) known as the “stop words” are uninformative, and removing them helps the model to focus on the important words

### ML Algorithms

#### Transfer Learning Algorithms

##### BERT Algorithm

BERT [25] is a state-of-the-art deep learning algorithm used extensively for NLP tasks using a transformer architecture to learn contextual representations of words in a sentence. BERT scrutinizes the words and their relationships in the post to capture nuanced meanings and emotions to analyze the textual content of the posts (eg, posts expressing momentary depressive feelings).

##### BERT Family Algorithms

In this study, we also used several subtypes of the BERT algorithm, that is, A Lite BERT (ALBERT) [44], Robustly Optimized BERT Approach (RoBERTa) [45], and Distilled BERT (DistilBERT) [46], to investigate their performance in detecting momentary depressive feelings.

#### Traditional ML Algorithms

##### CNN Algorithm

The CNN [47] is a deep learning algorithm that has been widely used in various computer vision and, more recently, in text analysis tasks. In post classification, CNNs can be used to detect posts with particular content (eg, momentary depressive feelings) by learning a representation of the post’s text that is fed into an algorithm to make a detection [48,49].

##### Bidirectional Long Short-Term Memory

Bidirectional long short-term memory (BiLSTM) is a type of recurrent neural network that is particularly well suited for processing sequential data such as text [50]. Unlike traditional neural networks that process input sequences in only one direction, BiLSTMs process the input sequence in both forward and backward directions, enabling the network to capture contextual information from both past and future time steps. This capability results in improved performance on text mining tasks. In the context of post analysis, a BiLSTM can be trained on a large data set of posts to learn the contextual relationships between the words in a post and detect whether a post expresses momentary depressive feelings. The BiLSTM considers the order of words in a post and the relationships between them, enabling it to capture more complex patterns and relationships in the data compared to traditional feedforward neural network.

### Evaluation

To investigate and evaluate the performance of competing algorithms, a 10-fold cross-validation (CV) approach is carried out. This involved randomly partitioning the input data into 2 sets: a CV set (2944/3680, 80% labeled posts) and a test set (736/3680, 20% labeled posts). The CV set was further divided into 10 subsets, allowing us to construct and train 10 distinct models. These models were subsequently evaluated using unseen data. The use of unseen data in CV is crucial for assessing the generalization capability of the models. This evaluation with unseen data helps mitigate the risk of overfitting and provides a more reliable estimation of the algorithm's performance in real-world scenarios [51]. The algorithm’s overall performance was computed by averaging the results obtained from the 10 runs. To assess the performance of competing algorithms to accurately identify posts expressing momentary depressive feelings, 6 evaluation metrics were calculated: area under the curve (AUC), accuracy, sensitivity, specificity, precision, and *F*_1_-score [52].

### Hyperparameter Sensitivity

To optimize algorithm performance, the tuning of hyperparameters emerges as a necessity. Yet, it is important to recognize that a universal approach for hyperparameter selection is not known to exist. In light of this, this study took a multifaceted approach by delving into distinct sets of hyperparameter values for each algorithm. This approach enabled us to carefully identify the configuration that attains high performance. A summary of contextual approaches and their respective hyperparameters used in this study can be found in Table 1.

**Table 1:** Hyperparameters for different contextual approaches.

Algorithm	Hyperparameters
BERT^a^, ALBERT^b^, RoBERTa^c^, and DistilBERT^d^	Learning rate (Adam): (5×10^–5^, 4×10^–5^, 3×10^–5^, 2×10^–5^, and 1×10^–5^) Batch size: (8, 16, 32, and 64) Training epochs: (2, 3, 4, 5, 6, and 7)
CNN^e^	Learning rate: (1×10^–4^, 1×10^–3^, and 1×10^–2^) Kernel size: (2, 3, 4, 5, and 6) Batch size: (16, 32, 64, and 128) Training epochs: (2, 3, 4, 5, 6, and 7)
BiLSTM^f^	Batch size: (16, 32, 64, and 128) Training epochs: (2, 3, 4, 5, 6, and 7)

^a^BERT: Bidirectional Encoder Representations From Transformers.

^b^ALBERT: A Lite BERT.

^c^RoBERTa: Robustly Optimized BERT Approach.

^d^DistilBERT: Distilled BERT.

^e^CNN: convolutional neural network.

^f^BiLSTM: bidirectional long short-term memory.

### Ethical Considerations

In contrast to conventional research involving human participants, ethical guidelines pertaining to social media research propose that publicly accessible data (eg, posts publicly posted on X) can be used for research purposes without necessitating supplementary consent or ethics endorsement [53,54]. In this study, we did not interact and intervene with the users whose public posts were collected and analyzed user-generated posts. It is worth noting, however, that any potentially associated identifying personal information (eg, user IDs and URLs) has been carefully eliminated to uphold anonymity and safeguard the privacy of X users.

## Results

### Hyperparameter Sensitivity Analysis

In this study, various contextual language approaches were used, each with a range of hyperparameters tuned to achieve optimal performance. Multiple iterations of each algorithm were carried out using different hyperparameter configurations. The hyperparameter combinations that yielded the highest performance for each model are summarized in Table 2.

**Table 2:** Optimal hyperparameter configurations.

Algorithm	Hyperparameters			
	Learning rate (Adam)	Batch size	Training epochs	Kernel size
BERT^a^	3×10^–5^	16	3	N/A^b^
ALBERT^c^	2×10^–5^	16	4	N/A
RoBERTa^d^	1×10^–5^	32	3	N/A
DistilBERT^e^	2×10^–5^	16	3	N/A
CNN^f^	1×10^–3^	64	4	4
BiLSTM^g^	N/A	32	3	N/A

^a^BERT: Bidirectional Encoder Representations From Transformers.

^b^N/A: not applicable.

^c^ALBERT: A Lite BERT.

^d^RoBERTa: Robustly Optimized BERT Approach.

^e^DistilBERT: Distilled BERT.

^f^CNN: convolutional neural network.

^g^BiLSTM: bidirectional long short-term memory.

### Performance Assessment

In this study, we undertook both binary and multilabel classifications. Nevertheless, it is noteworthy that the outcomes of the multilabel classification (see Multimedia Appendix 1 for details) were not as encouraging as those achieved in the binary classification task. Our analysis revealed an interesting finding in the context of multilabel classification, namely, that posts expressing depressive feelings, regardless of their intensity or scale, pose a challenge for classification models. The complexity of these posts makes it challenging to achieve precise categorization since they encompass a range of emotional states that may not align neatly with predefined categories. This insight highlights the complexity of classifying nuanced sentiment, particularly in the context of depressive expressions.

The performance of different algorithms in detecting momentary depressive feelings with binary classification is presented in Table 3. Our results indicated that BERT and DistilBERT outperformed in momentary depressive feelings detection and achieved the highest values in almost all performance metrics with AUC values of 95.80% and 96.71%, respectively. Additionally, both algorithms demonstrated high accuracy (96.03% and 97.40%), sensitivity (96.22% and 97.57), specificity (95.83% and 97.22%), precision (95.96% and 97.30%), and *F*_1_-score (96.09% and 97.44%). The performance of traditional ML algorithms was relatively poor with the highest scores achieved by CNN (AUC: 84.81% and accuracy: 84.79%) and BiLSTM (AUC: 79.91% and accuracy: 79.86%). These findings indicated that the transfer learning algorithms performed significantly superior by a substantial margin. For instance, DistilBERT achieved an AUC value nearly 12% points higher than the highest AUC achieved by CNN (84.81%). These findings confirm the feasibility of this algorithm in detecting momentary depressive feelings highlighting the effectiveness of transfer learning algorithms in NLP tasks.

It is important to note that the transfer learning algorithms, especially DistilBERT and BERT, achieved high values in other performance metrics such as sensitivity, specificity, precision, and *F*_1_-score, in addition to overall accuracy. High sensitivity and specificity demonstrate that these algorithms were able to accurately identify posts with momentary depressive feelings while avoiding false positive and false negative predictions. The significant performance variation observed between BERT and its more lightweight counterpart, ALBERT, was an important finding of this study. ALBERT incorporates parameter-reduction techniques, which may impact its ability to capture intricate nuances within the data as effectively as BERT. Furthermore, we have closely examined potential disparities in pretraining strategies and fine-tuning procedures, seeking to identify any factors that might contribute to the observed divergence in performance. By elaborating on these architectural and procedural distinctions, we aim to provide a comprehensive understanding of the reasons underlying BERT’s superior performance over ALBERT. This analysis not only informs this study but also contributes to the broader discourse on the comparative strengths and limitations of these prominent language models.

**Figure 2:**
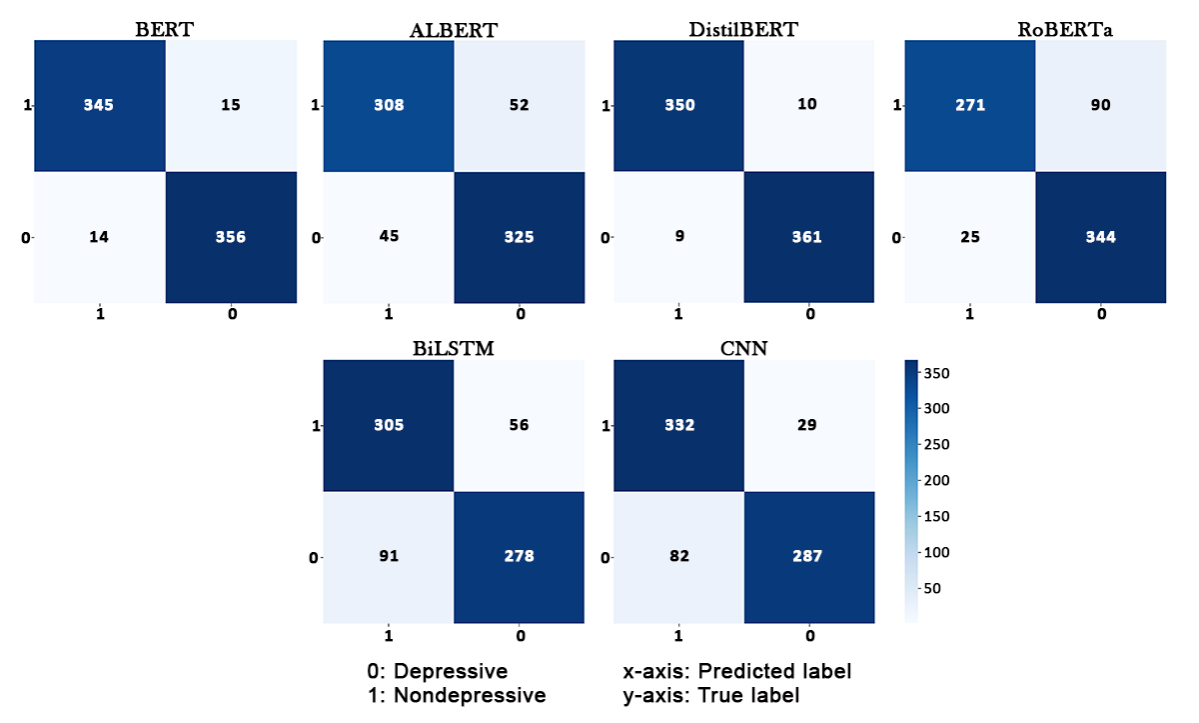
Confusion matrices produced by the competing algorithms for the test set. ALBERT: A Lite BERT; BERT: Bidirectional Encoder Representations From Transformers; BiLSTM: bidirectional long short-term memory; CNN: convolutional neural network; DistilBERT: Distilled BERT; RoBERTa: Robustly Optimized BERT Approach.

Overall, these results support the use of transfer learning algorithms for momentary depressive feelings detection in posts. Figure 2 depicts the class-wise results of competing algorithms using confusion matrices.

**Table 3:** The performance of different algorithms using post binary classification.

Algorithm	Performance metrics					
	AUC^a^ (%)	Accuracy (%)	Sensitivity (%)	Specificity (%)	Precision (%)	*F*_1_-score (%)
BERT^b^	95.80	96.03	96.22	95.83	95.96	96.09
ALBERT^c^	81.36	86.71	87.84	85.56	86.21	87.01
RoBERTa^d^	84.15	84.25	93.22	75.07	79.26	85.68
DistilBERT^e^	*96.71* ^f^	*97.40*	*97.57*	*97.22*	*97.30*	*97.44*
CNN^g^	84.81	84.79	77.78	91.97	90.82	83.80
BiLSTM^h^	79.91	79.86	75.34	84.49	83.23	79.09

^a^AUC: area under the curve.

^b^BERT: Bidirectional Encoder Representations From Transformers.

^c^ALBERT: A Lite BERT.

^d^RoBERTa: Robustly Optimized BERT Approach.

^e^DistilBERT: Distilled BERT.

^f^The best values for the performance metrics are in italics.

^g^CNN: convolutional neural network.

^h^BiLSTM: bidirectional long short-term memory.

## Discussion

### Principal Findings

This study aimed to detect momentary depressive feelings in X data using contextual language approaches. Our results indicated that (pretrained) transfer learning algorithms such as DistilBERT can effectively detect momentary depressive feelings with an accuracy of 97.4%. In this study, we used a comprehensive process of lexicon construction, data collection, and NLP-based ML algorithms to obtain accurate results. Our findings have practical implications in the mental health field by offering a potential framework for monitoring and detecting individuals’ mental health states in real time, which could facilitate timely interventions and support. For instance, this research can contribute to the development of automated systems that analyze social media posts, enabling mental health professionals to identify individuals expressing depressive feelings and subsequently provide support and resources.

The success of this study can be largely attributed to the effective use of a precise lexicon that contained a list of key terms relevant to depressive feelings based on prior research. These key terms were then manually evaluated to ensure their accuracy and relevance. The high intercoder reliability achieved by the researchers is a testament to the quality of the post labeling. This approach fostered the quality of the lexicon, leading to the accurate detection of momentary depressive feelings. Furthermore, the manual labeling of posts also contributed to the accuracy of the study because automatic labeling typically introduces noise into data and degrades algorithm performance [55].

In this study, we used a range of algorithms, including BERT, ALBERT, RoBERTa, DistilBERT, CNN, and BiLSTM. Notably, DistilBERT outperformed the other algorithms in detecting momentary depressive feelings. The use of multiple algorithms allowed for a comprehensive evaluation of the effectiveness of various algorithms in detecting momentary depressive feelings. These findings, consistent with previous studies, highlight the superiority of transfer learning algorithms in NLP tasks, particularly in the detection of momentary depressive feelings in social media data. This study used state-of-the-art algorithms that leverage large amounts of data and generalize to new tasks. Transfer learning algorithms are especially adept at processing large data sets and can identify patterns and features that are difficult to capture with traditional ML algorithms.

Within the domain of binary and multilabel classification, our analysis uncovers a compelling revelation. Specifically, our findings highlight the intricate nature of posts conveying depressive emotions, irrespective of their varying degrees or gradations. These posts present a formidable obstacle for classification models due to their nuanced character, encompassing a broad range of emotional states that defy simple categorization. This insight serves as a poignant reminder of the challenges inherent in accurately classifying such nuanced sentiments, especially when addressing the realm of depressive expressions.

The significant findings of this study have the potential to make a meaningful impact on mental health, particularly in momentary depressive feelings detection. Early detection of momentary depressive feelings can pave the way for timely interventions and ultimately improve mental health outcomes. The approach presented in this study could be integrated into social media monitoring tools to identify individuals who are at risk of developing depression or who may benefit from mental health interventions. This approach could lead to more efficient and effective mental health interventions, resulting in better outcomes for individuals with mental health conditions and reducing the burden imposed by mental health disorders. The findings of this study provide a beneficial stepping stone for the development of new and innovative approaches to mental health monitoring and intervention.

Although the findings of the study are promising, there are limitations. First, the study only focused on momentary depressive feelings, and the results may not be generalizable to other mental health conditions such as anxiety, stress, or other mood disorders. Second, the study relied solely on X data, which may not represent the broader population. Future research could investigate the use of other social media platforms or clinical data to assess the effectiveness of contextual language approaches for detecting mental health conditions in a more diverse population. Additionally, these approaches may be limited in their ability to capture the nuances and complexity of mental health conditions. Although this method is effective, it may not always detect posts that express subtle or indirect signs of mental health conditions. Future studies could explore the use of ML techniques to learn from the data to detect momentary depressive feelings rather than relying on predefined lexicons. This approach could lead to more accurate results and more effective detection of mental health conditions. Finally, the study only used English posts, which further limits the generalizability of the findings.

### Conclusions

This study aimed to detect momentary depressive feelings using X data and contextual language approaches. In this study, we applied a methodology consisting of data collection, manual labeling, and post analysis with contextual language approaches. A lexicon containing 32 keywords relevant to depressive feelings was established, and then, using this lexicon, *Twint* was used to extract posts from January 2022 to December 2022. Six baseline algorithms were used for the detection of momentary depressive feelings, and the results were evaluated using AUC, accuracy, sensitivity, specificity, precision, and *F*_1_-score.

Our results showed that DistilBERT, a transfer learning algorithm, had the highest performance in terms of the evaluation metrics described. The study found that transfer learning algorithms are promising tools in NLP tasks, for example, extracting knowledge and detecting patterns in posts, particularly in the detection of momentary depressive feelings.

Our findings demonstrated X data can be used for the detection of momentary depressive feelings. This is achieved through the development of an automated framework for continuously monitoring and detecting individuals’ real-time mental states. These findings have significant implications for timely mental health interventions. Early detection of momentary depressive feelings can prevent the escalation of these feelings to more severe depressive symptoms and reduce the burden imposed on people and society. This methodology can be easily applied to large X data sets, making it a useful tool for monitoring depressive symptoms on a large scale. Moreover, this methodology can be improved to be applied to other social media platforms and various mental health conditions. Overall, this study contributes to the growing body of research on using social media data for mental health research. Our approach provides a useful tool for researchers interested in studying momentary depressive feelings using social media data.

## Appendix

Multimedia Appendix 1 Evaluation performance for multilabel classification.

## Abbreviations

ALBERT: A Lite BERT
AUC: area under the curve
BERT: Bidirectional Encoder Representations From Transformers
BiLSTM: bidirectional long short-term memory
CNN: convolutional neural network
CV: cross-validation
DistilBERT: Distilled BERT
ML: machine learning
NLP: natural language processing
RoBERTa: Robustly Optimized BERT Approach
VADER: Valence Aware Dictionary for Sentiment Reasoning

## Data Availability

The data sets generated during and/or analyzed during this study are available from the corresponding author on reasonable request.
