# Supplementary material for: Momentary Depressive Feeling Detection Using X (Formerly Twitter) Data: Contextual Language Approach
Source: JMIR AI. 2023 Nov 27;2:e49531. doi: 10.2196/49531 (PMC11041470; doi:10.2196/49531)
Supplement: Multimedia Appendix 1 [file ai_v2i1e49531_app1.docx]

**Multimedia Appendix 1.** Evaluation performance for multilabel classification.

To assess the effectiveness of our models in correctly identifying the class of 5-scale posts within our dataset, we conducted multi-label classification experiments. The outcomes reveal that the performance of competing algorithms is not as encouraging as that observed in binary classification scenarios. In this experiment, the algorithm with the highest performance achieved values for all metrics that were lower than those achieved in binary classification. As highlighted in Table S1, the DistilBERT algorithm achieved the highest area under the curve (AUC) (77.17%), but it was below the AUC achieved by the poorest algorithm in the binary classification context. Additionally, of all the multi-label classification experiments, the DistilBERT algorithm achieved the highest accuracy (76.58%), sensitivity (73.38%), specificity (68.17%), precision (79.04%), and F_1_-score (70.18%), which again were all lower than those achieved by the poorest algorithm in binary classification.

| **Table S1.** The performance of different algorithms for post multi-label classification | | | | | | |
| --- | --- | --- | --- | --- | --- | --- |
| **Algorithm** | **Performance Metrics** | | | | | |
|  | **AUC^a^ (%)** | **Accuracy (%)** | **Sensitivity (%)** | **Specificity (%)** | **Precision (%)** | **F_1_-score (%)** |
| BERT^b^ | 76.80 | 76.17 | 71.87 | 66.10 | 76.00 | 75.93 |
| ALBERT^c^ | 74.32 | 73.85 | 73.17 | 68.08 | 73.71 | 74.63 |
| RoBERTa^d^ | 74.14 | 75.21 | 75.62 | 67.28 | 74.26 | 5.68 |
| DistilBERT^e^ | *77.17*^f^ | *76.58* | *73.38* | *68.17* | *79.04* | *70.18* |
| CNN | 63.15 | 63.92 | 59.53 | 61.51 | 63.34 | 64.63 |
| BiLSTM | 61.72 | 62.23 | 58.98 | 59.47 | 62.34 | 62.08 |
| ^a^AUC: area under the curve.  ^b^BERT: Bidirectional Encoder Representations From Transformers.  ^c^ALBERT: A Lite BERT  ^d^RoBERTa: Robustly Optimized BERT Approach  ^e^DistilBERT: Distilled BERT  ^f^The best values for the performance metrics are in italics. | | | | | | |

Our results showed that irrespective of the extent of their depressive contents, posts present a formidable hurdle for multi-label classification models. The multifaceted nature of these messages introduces intricacies that complicate the task of achieving precise categorization, mainly due to their diverse range of emotional nuances that defy conventional classification boundaries. This challenge is particularly amplified when dealing with expressions of depression, where the subtle shifts in language and sentiment can elude rigid predefined categories. The endeavor to accurately classify such complex emotional states presents a formidable obstacle because these expressions often defy straightforward classification due to their amalgamation of sentiments. Despite the substantial difficulty in achieving high classification accuracy within this specific domain, our study yields invaluable insights into the constraints and prospective pathways for enhancing classification within this specific domain. Through our investigation, we have illuminated the limitations inherent to existing multi-label classification models when applied to nuanced sentiment analysis, especially in the realm of depressive expressions. By recognizing these limitations, we pave the way for the application of binary classification that could potentially refine classification performance. For completeness, Figures S1-S6 show the class-wise confusion matrices for competing algorithms.


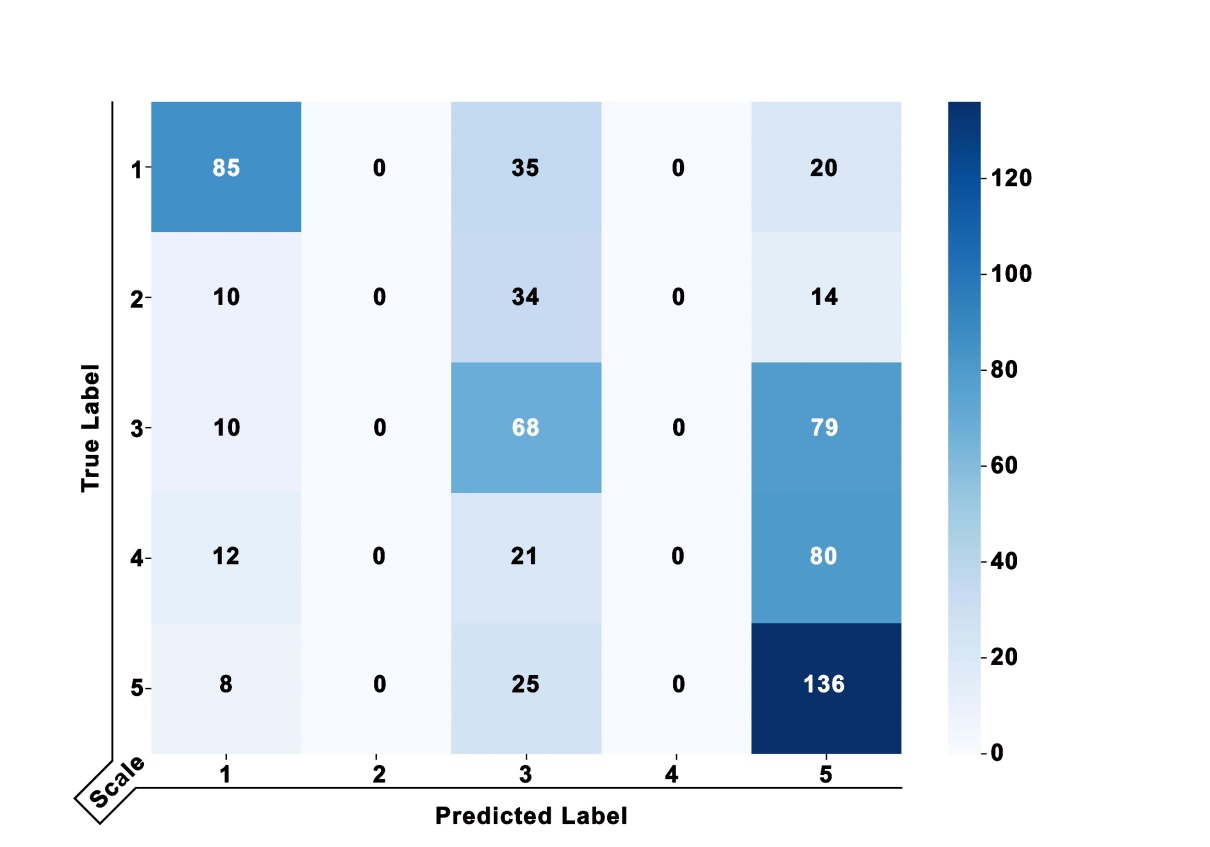


**Figure S1.** Multi-label confusion matrix produced by the BERT (Bidirectional Encoder Representations From Transformers) algorithm. Posts on a 5-point scale, with 5 indicating significant relevance to momentary depressive feelings.


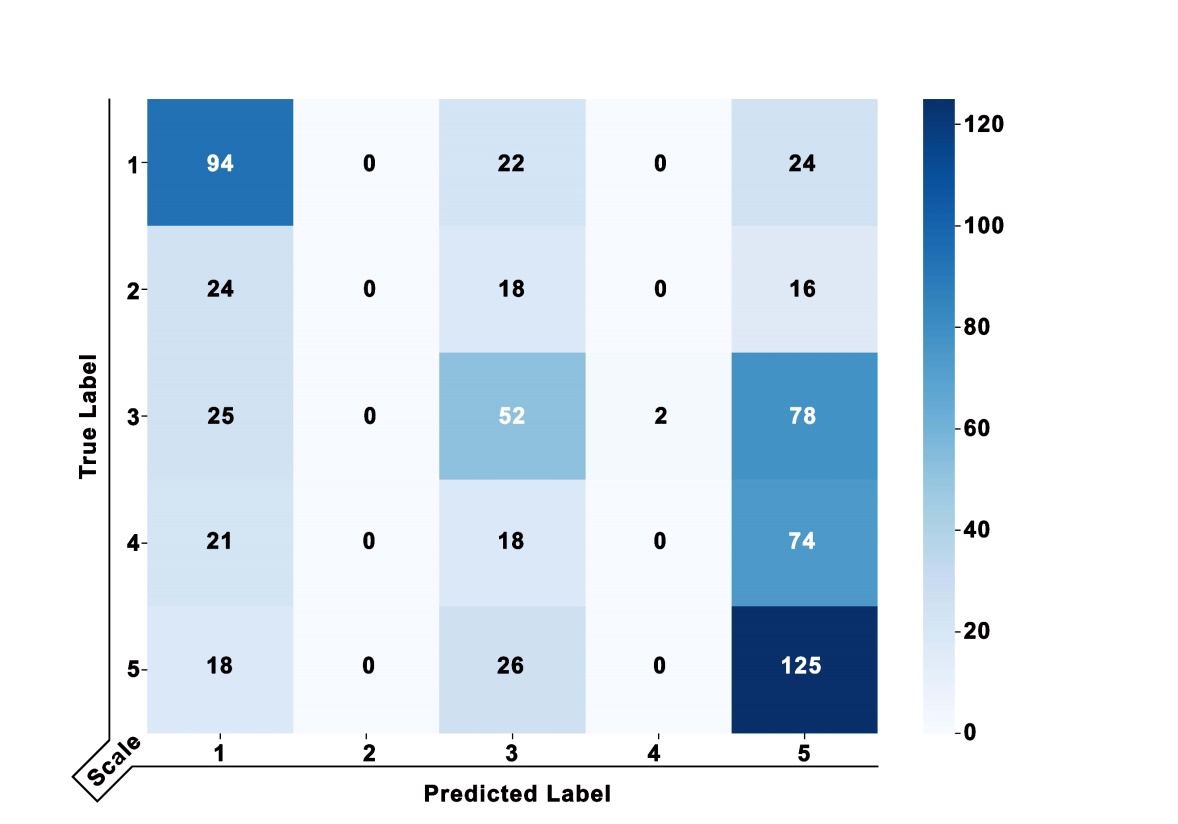


**Figure S2.** Multi-label confusion matrix produced by the DistilBERT (Distilled BERT) algorithm.

Posts on a 5-point scale, with 5 indicating significant relevance to momentary depressive feelings.


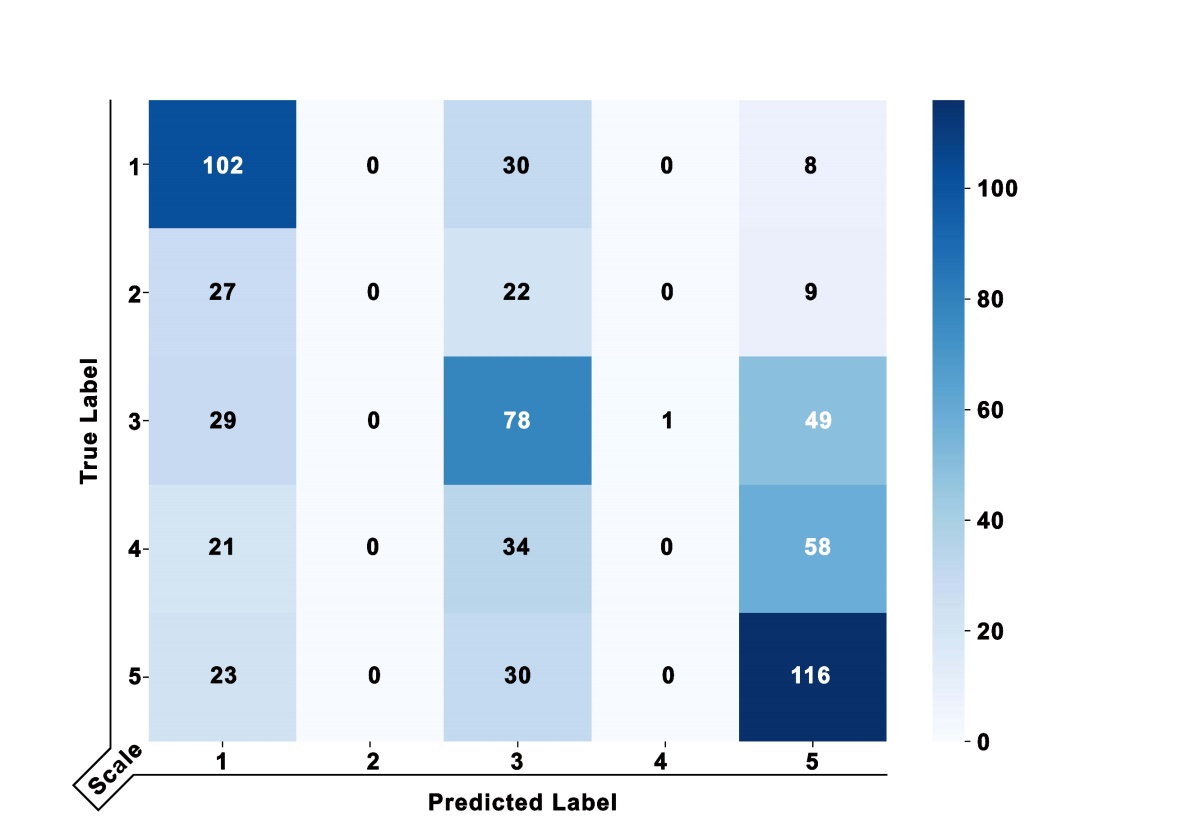


**Figure S3.** Multi-label confusion matrix produced by the ALBERT (A Lite BERT) algorithm.

Posts on a 5-point scale, with 5 indicating significant relevance to momentary depressive feelings.


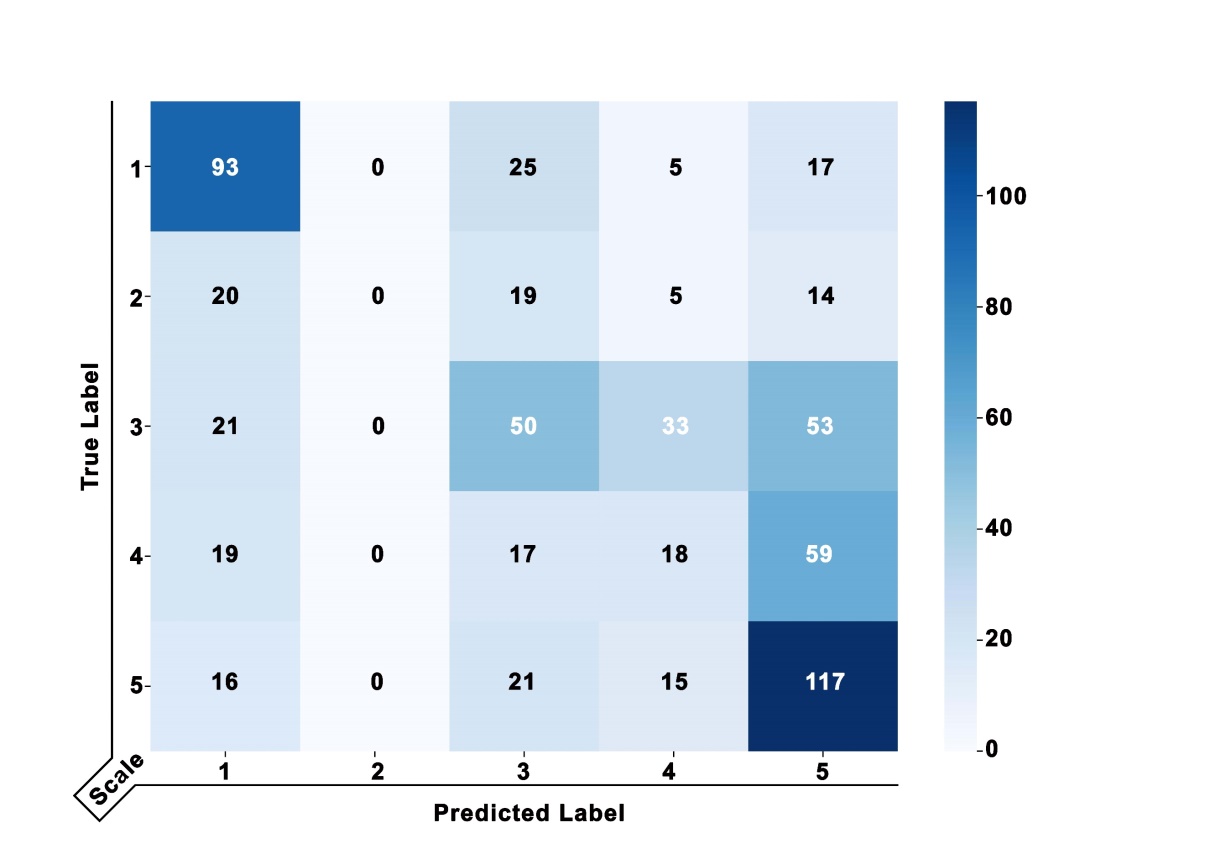


**Figure S4.** Multi-label confusion matrix produced by the RoBERTa (Robustly Optimized BERT Approach) algorithm.

Posts on a 5-point scale, with 5 indicating significant relevance to momentary depressive feelings.


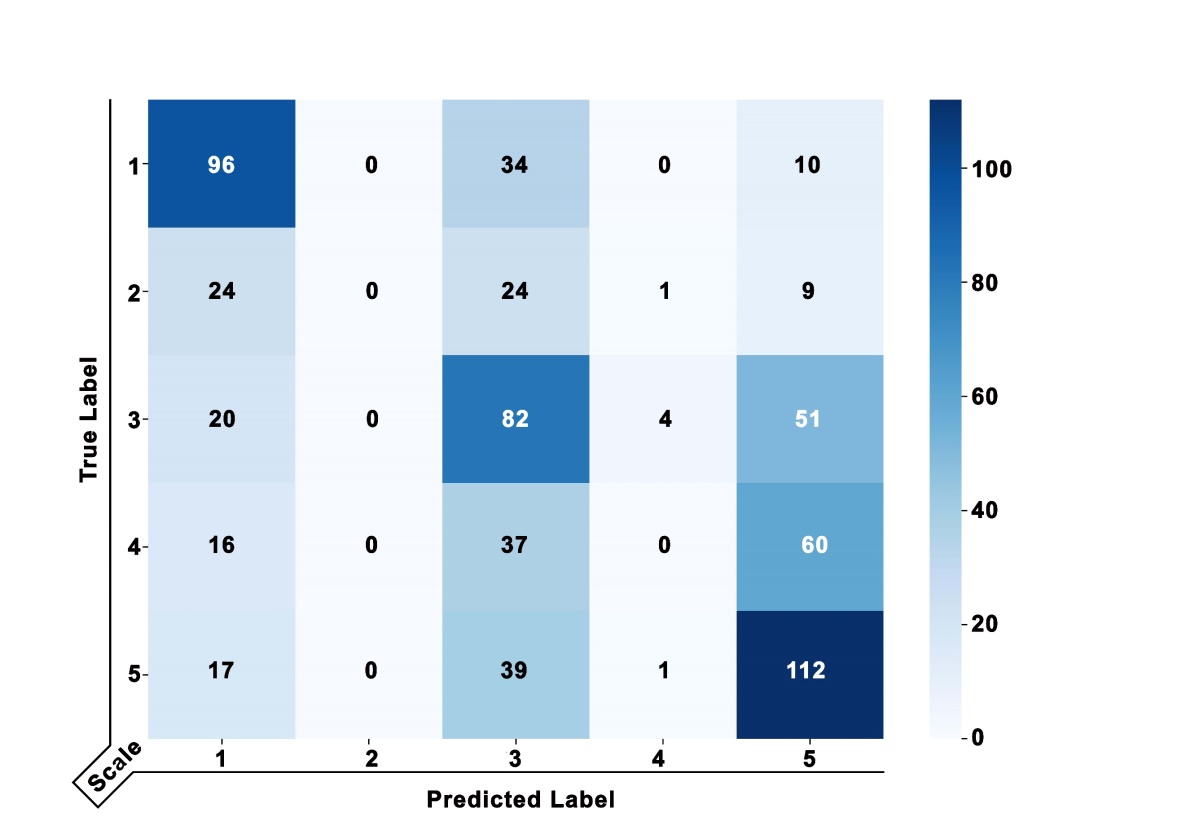


**Figure S5.** Multi-label confusion matrix produced by the CNN (Convolutional Neural Network) algorithm.

Posts on a 5-point scale, with 5 indicating significant relevance to momentary depressive feelings.


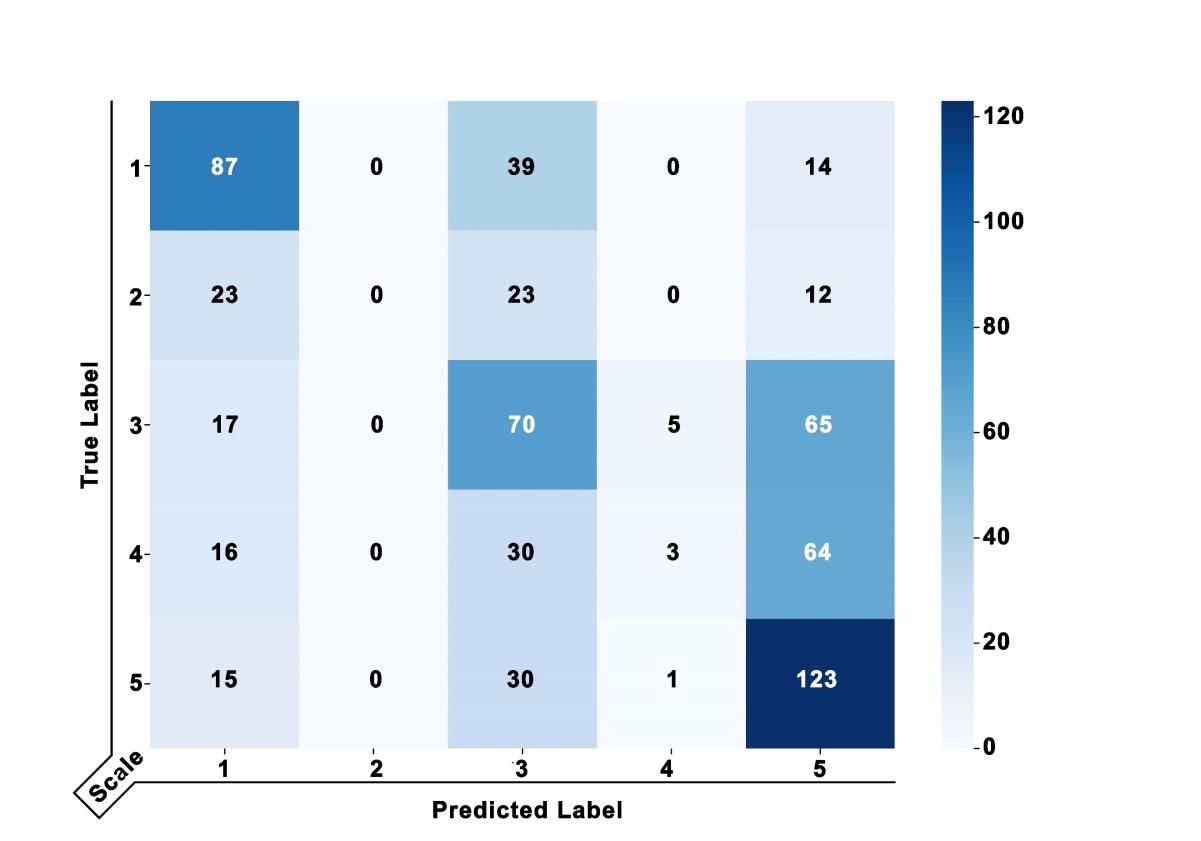


**Figure S6.** Multi-label confusion matrix produced by the BiLSTM (Bidirectional Long Short-Term Memory) algorithm.

Posts on a 5-point scale, with 5 indicating significant relevance to momentary depressive feelings.
